# Opportunities and risks of ChatGPT in medicine, science, and academic publishing: a modern Promethean dilemma

**DOI:** 10.3325/cmj.2023.64.1

**Published:** 2023-02

**Authors:** Jan Homolak

**Affiliations:** 1 Department of Pharmacology, University of Zagreb School of Medicine, Zagreb, Croatia; 2 Croatian Institute for Brain Research, University of Zagreb School of Medicine, Zagreb, Croatia

The release of ChatGPT, the latest large (175-billion-parameter) language model by San Francisco-based company OpenAI, prompted many to think about the exciting (and troublesome) ways artificial intelligence (AI) might change our lives in the very near future. The OpenAI's chatbot allegedly gained more than 1 million users in the first few days after its launch and 100 million in the first 2 months, positioning itself as the fastest-growing consumer application in history (1). The hype surrounding ChatGPT is not unjustified: the model is (still) free, easy to use, and able to authentically converse on many subjects in a way that is almost indistinguishable from human communication. Furthermore, considering that ChatGPT was generated by fine-tuning the GPT-3.5 model from early 2022 with supervised and reinforcement learning (2), the quality of the chatbot-generated content can only be improved with additional training and optimization. As the inevitable implementation of this disruptive technology will have far-reaching consequences for medicine, science, and academic publishing, we need to discuss both the opportunities and risks of its use.

## Can ChatGPT replace physicians?

AI has a tremendous potential to revolutionize health care and make it more efficient by improving diagnostics, detecting medical errors, and reducing the burden of paperwork (3,4); however, chances are it will never replace physicians. Algorithms perform relatively well on knowledge-based tests despite the lack of domain-specific training; ChatGPT achieved ~ 66% and ~ 72% on Basic Life Support and Advanced Cardiovascular Life Support tests, respectively (5), and performed at or near the passing threshold on the United States Medical Licensing Exam (6,7). However, they are notoriously bad at context and nuance (8) – two things critical for safe and effective patient care, which requires the implementation of medical knowledge, concepts, and principles in real-world settings. In their analysis of the future of employment, Frey and Osborne estimate that, while the probability of administrative health care jobs automation is relatively high (eg, 91% for health information technicians), the probability of automating the jobs of physicians and surgeons is 0.42% (9). While we might object as some evidence indicates that fully autonomous robotic systems might be “just around the corner“ (10), the job of a surgeon goes far beyond performing a surgical procedure. The complexity of the physician's job lies in the ability to administer fully integrated care by providing treatment but also compassion. As medical students we were taught to always take care of patients and not of their medical records – a clinical skill that computer algorithms are still not able to comprehend. Therefore, the tremendous potential of AI in healthcare does not lie in the possibility of replacing physicians, but rather in the capacity to increase physicians’ efficacy by redistributing workload and optimizing performance. In the words of Alvin Powell from The Harvard Gazette, „*A properly developed and deployed AI, experts say, will be akin to the cavalry riding in to help beleaguered physicians struggling with unrelenting workloads, high administrative burdens, and a tsunami of new clinical data.*“ (11).

There are also some ethical issues to consider regarding conversational AI in medical practice. Training a model requires a tremendous amount of (high-quality) data, and current algorithms are often trained on biased data sets. In fact, the models are not only susceptible to availability, selection, and confirmation bias but are also unreluctant to amplify it (12). For example, ChatGPT can provide biased outputs and perpetuate sexist stereotypes (13) – a challenge that has to be resolved before similar AI can be successfully and safely implemented in clinical practice (14-17). Other ethical issues are related to the legal framework. For example, it remains to be determined who is to blame when an AI physician makes an inevitable mistake.

## A chatbot-scientist

ChatGPT already wrote essays, scholarly manuscripts, and computer code, summarized scientific literature, and performed statistical analyses (18,19). Furthermore, AI might soon be able to successfully perform more complex assignments such as designing experiments (20) or conducting a peer-review (18). In some of the mentioned tasks, ChatGPT performed alarmingly well. In a recent experiment, researchers used existing publications to generate 50 research abstracts that were able to pass the plagiarism check performed by a plagiarism checker, an AI-output detector, and human reviewers (21). On the one hand, the astounding ability of ChatGPT to write specialized texts suggests that similar tools might soon be able to write complete research manuscripts, which would enable scientists to focus on designing and performing the experiments rather than on writing manuscripts (18). The latter might promote quality and equity in research by shifting the focus from the presentation to the content and experimental results. On the other hand, conversational AIs are just language models trained to sound convincing, but without the ability to interpret and understand the content. Consequently, ChatGPT-generated manuscripts might be misleading, based on non-credible or completely made-up sources (18). The worst part is, the ability of ChatGPT to write a text of surprising quality might deceive reviewers and readers, with the final result being an accumulation of dangerous misinformation. StackOverflow, a popular forum for computer programming-related discussions, banned the use of ChatGPT-generated text “*because the average rate of getting correct answers from ChatGPT is too low, the posting of answers created by ChatGPT is substantially harmful to the site and to users who are asking and looking for correct answers*“ (22). ChatGPT seems to be equally unreliable when it comes to writing research articles. For example, Blanco-Gonzalez et al assessed the ability of ChatGPT to assist human authors in writing review articles and concluded that “…*ChatGPT is not a useful tool for writing reliable scientific texts without strong human intervention. It lacks the knowledge and expertise necessary to accurately and adequately convey complex scientific concepts and information.*” (23). On top of that, the chatbot seems to have an alarming tendency to make up references with the goal of sounding convincing (18,24,25). In fact, the creators of ChatGPT openly disclosed that the fact that “*ChatGPT sometimes writes plausible-sounding but incorrect or nonsensical answers*” a “challenging issue to fix“ (2). A failure to acknowledge the limitations of conversational AI might pose an additional strain on the publishing system already flooded with meaningless data and low-quality manuscripts. Apart from the problem of unreliability, there are several additional ethical challenges (18,19,26). A chatbot cannot be held accountable for its work, and there is no legal framework to determine who owns the rights to the AI-generated work – the author of the manuscript, the author of the AI, or the (unknown) authors who contributed training data? Furthermore, since ChatGPT often fails to disclose the source of information, who is to blame for plagiarism if the chatbot decides to plagiarize? Until the ethical dilemmas are resolved, most publishers agree that the use of any kind of AI should be clearly acknowledged and that chatbots should not be listed as authors.

## Where do we go from here?

The powerful disruptive technology of conversational AIs is here to stay, and we can only expect them to improve with additional training and optimization. Banning or actively ignoring their use makes no sense – they can dramatically improve many aspects of our lives by alleviating the burden of daunting and repetitive tasks. In medicine, AI might dramatically improve efficacy just by alleviating a fragment of the suffocating paperwork (27), and optimized chatbots (eg, Stanford's BioMedLM) (28) might speed up and improve literature search. Nevertheless, we should not be allured by the overwhelming potential of AI. For AI to realize its full potential in medicine and science, we should not implement it hastily but advocate its mindful introduction and an open debate about the risks and benefits.
